# Influence of Structural Optimization on the Physical Properties of an Innovative FDM 3D Printed Thermal Barrier

**DOI:** 10.3390/ma17246293

**Published:** 2024-12-23

**Authors:** Beata Anwajler, Jacek Iwko, Anna Piwowar, Roman Wróblewski, Piotr Szulc

**Affiliations:** 1 Department of Energy Conversion Engineering, Faculty of Mechanical and Power Engineering, Wroclaw University of Science and Technology, 27 Wybrzeze Wyspianskiego Street, 50-370 Wroclaw, Poland; a.piwowar@pwr.edu.pl (A.P.); piotr.szulc@pwr.edu.pl (P.S.); 2 Faculty of Mechanical Engineering, Wroclaw University of Science and Technology, 27 Wybrzeze Wyspianskiego Street, 50-370 Wroclaw, Poland; jacek.iwko@pwr.edu.pl (J.I.); r.m.wroblewski@pwr.edu.pl (R.W.)

**Keywords:** thermal insulation, additive manufacturing, 3D printing, fused deposition modeling, PLA, Kelvin cells, mechanical properties, thermal properties

## Abstract

This article describes an innovative thermal insulation barrier in the form of a sandwich panel manufactured using 3D FDM printing technology. The internal structure (core structure) of the barrier is based on the Kelvin foam model. This paper presents the influence of the parameters (the height h and the porosity P of a single core cell) of the barrier on its properties (thermal conductivity, thermal resistance, compressive strength, and quasi-static indentation strength). The dominant influence of the porosity of the structure on the determined physical properties of the fabricated samples was demonstrated. The best insulation results were obtained for single-layer composites with a cell height of 4 mm and a porosity of 90%, where the thermal conductivity coefficient was 0.038 W/(m·K) and the thermal resistance 0.537 (m^2^·K)/W. In contrast, the best compressive strength properties were obtained for the 50% porosity samples and amounted to about 350 MPa, while the moduli for the 90% porosity samples were 14 times lower and amounted to about 26 MPa. The porosity (P) of the composite structure also had a significant effect on the punch shear strength of the samples produced, and the values obtained for the 90% porosity samples did not exceed 1 MPa. In conclusion, the test showed that the resulting 3D cellular composites offer an innovative and environmentally friendly approach to thermal insulation.

## 1. Introduction

Currently, there is a significant increase in global energy demand. While the share of renewable energy sources in the global market is steadily growing, fossil fuels remain the primary source of energy worldwide [1]. As energy demand rises, non-renewable resources are being depleted. Furthermore, their processing results in carbon dioxide (CO_2_) emissions and is a major contributor to environmental degradation [2].

Due to the aforementioned reasons, there is an increasing emphasis on the need to implement the concept of sustainable development, which advocates for the prudent use of resources [3]. Achieving this goal requires wise energy management, including reducing consumption and improving efficiency [4,5]. One method to minimize energy losses is the use of structures that provide high insulation efficiency.

The development of modern, sustainable thermal insulation barriers should consider their environmental impact and the use of raw materials from which they are produced. The production process should consume as little energy as possible, generate minimal post-production waste, or ensure that waste is recycled [6]. These conditions can be met using 3D printing techniques, which offer numerous environmental benefits compared to conventional production methods. They generate less material waste, and the production processes are characterized by lower energy consumption and reduced greenhouse gas emissions [7]. 3D printing allows for manufacturing from a wide range of materials. Reusable construction materials, such as plastic products based on recycled polymers and metal waste, are available [8]. Additionally, 3D printing enables the creation of complex geometries that would be difficult to achieve using conventional manufacturing techniques [9]. This provides the ability to control the internal geometry of printed barrier structures, thereby optimizing their thermo-mechanical properties.

Sola et al. [10], based on a literature review, indicate that FDM 3D printing, in comparison to other manufacturing methods, is characterized by efficient use of construction materials, reduced transportation needs due to the possibility of decentralized production, and the potential for using recycled filaments. However, one of the main environmental disadvantages of FDM and other 3D printing methods is the significant energy consumption of the printers. The authors noted that there is potential to reduce energy consumption in FDM printers by reducing printing time, nesting multiple parts in a single print job, increasing layer height and reducing infill density. Furthermore, the authors highlighted that the environmental impact of FDM largely depends on the source of the electricity used, suggesting that it can be reduced by transitioning to renewable energy sources. Babagowda et al. [11] investigated the mechanical properties (tensile strength, flexural strength) of specimens fabricated using 3D printing FDM technology from PLA material blended with recycled PLA, in various mass ratios of recycled PLA to virgin PLA (ranging from 10% to 50%). The study revealed that recycled PLA retains its mechanical properties to some extent, allowing its application in less-demanding use cases. Furthermore, the authors emphasized the necessity of optimizing printing parameters to achieve satisfactory mechanical properties, noting that printing with a lower layer height enhances the mechanical performance of printed objects. Patti et al. [12] characterized two types of commercial PLA filaments—virgin and recycled—using infrared spectroscopy (IR), thermogravimetric analysis (TGA), dynamic rheology, and dynamic mechanical analysis (DMA) of the material after extrusion. For the recycled material, a slight reduction in the ATR spectra was observed relative to typical PLA polymer absorption bands. However, no significant differences were identified in terms of thermal degradation, rheological behavior, and thermo-mechanical properties between the recycled and virgin PLA filaments.

In recent years, many research efforts have been carried out to improve traditional and develop novel thermal insulation materials for increased energy efficiency and reduced negative environmental impact. Aerogels are an example of novel, constantly researched thermal insulation materials. Shi et al. [13] improved the thermal insulating properties of SiO_2_ aerogels by optimizing the sol-gel process, obtaining aerogels with ultra-low thermal conductivity in the range of 0.0135 to 0.021 W/(m⸱K). Ghica et al. [14] reinforced silica aerogels with aramid nanofibers and microfibers, obtaining materials with a thermal conductivity of 0.0383 W/(m⸱K). In addition, they showed that by using nanofibers to reinforce aerogel nanocomposites, it is possible to improve their mechanical properties (increase in stiffness and strength). A recent trend is the search for the use of waste materials in thermal insulation. Ali et al. [15] fabricated thermal insulation and sound absorption composites from waste black tea bags and date palm tree surface fibers with thermal conductivity lower than 0.07 W/(m⸱K) and noise reduction coefficients above 0.37. Malet-Damour et al. [16] proved that loose-fill plastic waste (LFPW) can be used as effective, economically viable and sustainable barriers to thermal insulation. Marín-Calvo et al. [17] analyzed an insulating material based on waste materials—cellulose and rice husks. The material developed by the authors had a thermal conductivity coefficient of 0.04 W/(m⸱K) and a compressive strength ranging from 20.19 to 21.23 MPa. Rojas et al. [18] produced thermal insulation materials based on agricultural residual wheat straw and corn husk biomass with thermal conductivities in the range of 0.046 to 0.047 W/(m⸱K). Several researchers have focused on the application of phase change materials (PCMs) in thermal insulation. Zhang et al. [19] investigated the thermal performance of lightweight concrete masonry unit (CMU) blocks (bricks) in which the voids were filled with extruded polystyrene (XPS) and/or based on dry powder microparticles-based phase change material (PCM) with a paraffin-containing a silica-based matrix. Studies have demonstrated that filling blocks with XPS and/or PCM may improve their thermal performance. In addition, under the appropriate conditions, a higher increase in thermal performance may be achieved with used phase change materials than with XPS. PCM materials have a high energy storage density through which their use in construction can facilitate the creation of energy-efficient buildings [20,21,22].

The potential for using 3D printing in the manufacturing of thermal insulation barriers has only recently begun to be analyzed. Gama et al. [23] noted that although the production of 3D printed foams had already been extensively studied, the main emphasis in reports was placed on their mechanical or their cushion behavior. Therefore, the researchers focused on determining the thermal insulation properties of foams printed using FDM technology from polyurethane/cork filaments. The conducted tests determined that the printed polyurethane/cork foams may be effectively used as thermal insulation barriers, which is confirmed by the thermal conductivity values obtained from thermal tests, ranging from 0.044 W/(m⸱K) to 0.049 W/(m⸱K). Islam et al. [24] investigated panels with a porous internal structure fabricated using FDM printing technology from PLA filament. The research indicated that the developed panels offered very high potential for commercial use as thermal insulation barriers for buildings, basing this conclusion on the thermal tests, according to which thermal conductivity values for the panels range from 0.037 W/(m⸱K) to 0.070 W/(m⸱K). De Rubeis et al. [25,26,27] proved that the complexity level of the internal structure of blocks printed by FDM technology using PLA filament can significantly affect their thermal insulating abilities. Their research suggests that among the internal structures analyzed, the most complex honeycomb structure provided the highest insulating properties, and the resulting thermal transmittance value was 1.22 W/(m^2^⸱K). In addition, the effect of using waste thermal insulation materials, such as polystyrene, wood sawdust, sheep’s wool, and hemp, on the thermal insulation efficiency of the blocks was analyzed. Research confirmed that infill of the voids of honeycomb blocks with waste materials reduces the thermal transmittance value and therefore improves thermal insulation properties. The highest level of thermal transmittance reduction was achieved by using sheep’s wool as filler. Thus, the thermal transmittance value was reduced to 0.53 W/(m^2^⸱K), a reduction of about 57%.

As thermal insulation barriers, cellular materials are commonly used. These materials utilize a structure containing pores to achieve desired properties. The thermal insulating properties of cellular materials are primarily influenced by the low thermal conductivity of the gas within the pores but also by thermal radiation and gas convection, which are affected by the distribution of the solid phase surrounding the pores [28,29]. Thus, the heat transfer through cellular material is an extremely complex phenomenon influenced by factors such as the material’s internal structure. Differences in the internal structure of cellular material impact not only its thermal properties (such as thermal conductivity, thermal resistance) but also its mechanical properties, such as compressive strength and quasi-static indentation strength. Each of these properties affects the potential use of barriers with a cellular structure for thermal insulation applications.

Determining the impact of a thermal insulation barrier’s cellular core structure requires the implementation of a model that defines it. One method to describe the structure of cellular materials is the Kelvin model. This model assumes that the best approximation of a foam can be obtained by the replication of a single cell shaped as a tetrakaidecahedron with slightly curved faces [30].

The aim of this paper is to develop an innovative thermal insulation barrier based on a Kelvin foam model, fabricated using 3D FDM printing technology, and to experimentally determine the thermo-mechanical properties of the barrier (thermal conductivity, thermal resistance, compressive strength, and quasi-static indentation strength) as a function of the variables determining its internal geometry. The materials under investigation were produced using polylactic acid (PLA), which is characterized by biodegradability, non-toxicity, biological origin, and a low melting temperature (150–160 °C) [31,32]. PLA is the dominant biodegradable polymer for 3D printing due to its ease of processing, mechanical strength, availability, and cost [33]. Despite the lower thermal resistance of polylactic acid (PLA) compared to acrylonitrile-butadiene-styrene (ABS), PLA offers better strength and stiffness, which is a key factor in determining mechanical properties [34]. PLA is not very sensitive to temperature changes. PLA is also environmentally friendly as it is made from renewable resources such as corn and potato peels.

## 2. Materials and Methods

### 2.1. Research Object

**Figure 1:**
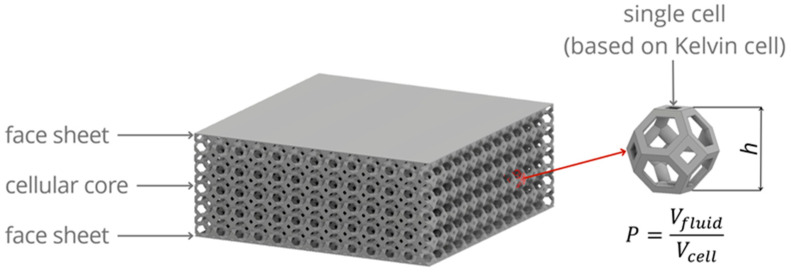
Concept sketch of the thermal insulation barrier.

The research concerns an innovative thermal insulation barrier in the form of a sandwich panel with an open-cell core, the structure of which consists of modified Kelvin cells, as shown in Figure 1.

The geometry of a single core cell is determined by two variables—height h and porosity, defined as P = V_fluid_/V_cell_ or P = 1−V_solid_/V_cell_, where V_fluid_ is the volume of fluid (air) in the total volume of the cell V_cell_, while V_solid_ is the volume of solid (core matrix) in the total volume of the cell V_cell_. The total volume of a single core cell is the volume of a truncated octahedron; thus, it is formulated as V_cell_ = h^3^/2.

The height of the sandwich panel equal to 20 mm and the height of the face sheet equal to 1 mm were assumed. The ranges of analyzed cell porosities P and cell heights h were determined after a practical examination of the limitations of the specifications of the 3D printer used.

### 2.2. Research Design

In order to determine the minimum number of thermal insulation barrier variants that should be adopted for the experimental research, an experimental design was used. A two-factor three-level (3^2^) full factorial design was applied. Both independent variables (cell porosity P and cell height h) were examined at three levels, expressed as low (−1), medium (0), and high (1). The determined ranges of the independent variables and their coded values are presented in Table 1. The matrix of the design is presented in Table 2.

### 2.3. Sample Manufacturing Process

Commercial PLA filament of 1.75 mm diameter (ROSA 3D, Gdańsk, Poland) was used to manufacture samples in the Bambu Lab X1 Carbon Combo 3D printer. Autodesk Inventor was used to design the samples, and Bambu Studio was used to prepare the 3D printing models. In Table 3, the 3D printing parameters are listed.

Cell porosity P and cell height h were imparted to the analyzed samples at the virtual model creation stage, before printing.

**Figure 2:**
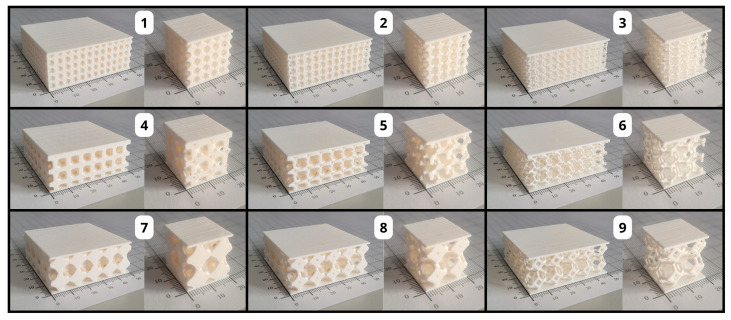
Printed test samples labeled with the experiment code.

In total, 6 copies of each of the 9 barrier variants from the experimental matrix with dimensions of 50 mm × 50 mm × 20 mm were printed to determine thermal conductivity, thermal resistance, and quasi-static indentation strength in 6 repetitions. In addition, 6 copies of each of the 9 barrier variants from the experimental matrix with dimensions of 50 mm × 50 mm × 20 mm were printed to determine compressive strength in 6 repetitions. A total of 108 samples were fabricated. Photos of the printed test samples with the experiment code marking are shown in Figure 2.

**Figure 3:**
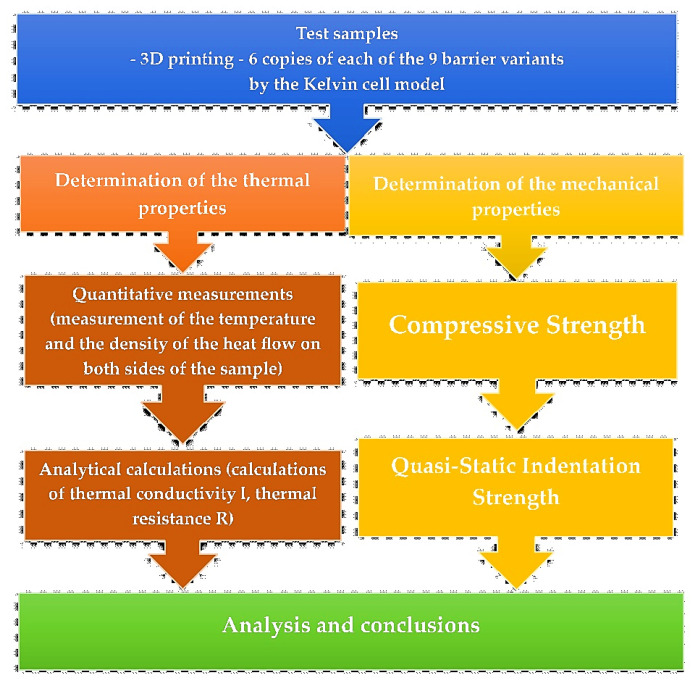
Methodological process used in this study.

The flowchart of the research procedure used is shown in Figure 3.

### 2.4. Determination of Properties

#### 2.4.1. Thermal Conductivity and Thermal Resistance

The thermal insulation properties, i.e., the coefficient of thermal conductivity *λ* and the thermal resistance R, were determined experimentally in accordance with ISO 9869-1:2014 [35] using a test relocated at the Department of Energy Conversion Engineering, Faculty of Mechanical and Power Engineering, Wroclaw University of Technology. A detailed description of the test rig and the method of determining and calculating the thermal properties is described in other publications by the co-author [36,37,38]. Measurements were performed on all the test specimens described above.

**Figure 4:**
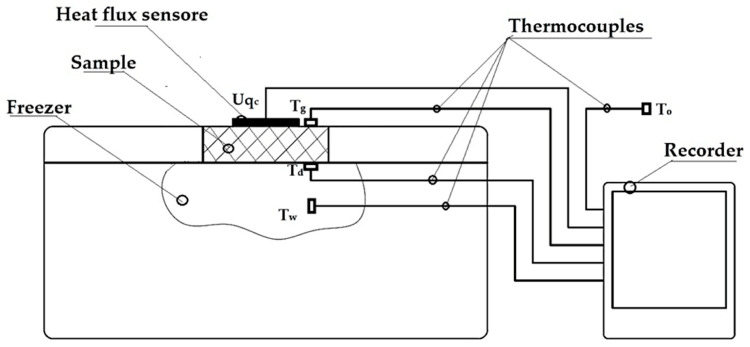
Schematic of the test stand for thermal insulation testing [36,37,38].

A schematic diagram of the test rig is shown in Figure 4.

During testing, the samples were placed in the lid opening of an Aisberg LP15 C15 freezer (MELIS, Poznań, Poland) so that the lower part of the samples was in direct contact with the inside of the freezer and the upper part was exposed to the outside. The process of heat transfer through the sample was based on the temperature difference between the environment (outside) and the center of the freezer. The intensity of heat flow through the insulation under test was determined using an FHF04SC sensor (Hukseflux Thermal Sensors B.V., Delft, The Netherlands), and data were recorded on the recorder every 0.5 min. Temperature measurements were taken at the following locations: on the outer surface of the sample, on the inner surface of the sample, inside the fridge/freezer, and around the outside of the fridge/freezer (location of thermocouples shown in Figure 3). Temperatures on the external surface of the sample were set at +20 °C on the ambient side and −20 °C inside the fridge/freezer, taking into account standard operating conditions for building insulation. Measurements were taken after thermal equilibrium had been reached, which was considered to have been reached when the temperature differences on the surface of the samples tested did not exceed 0.5 °C between successive measurements taken over a period of 1 h. The thermal insulation properties of the materials were determined at an average sample temperature of 0 °C. The results obtained were used to determine the thermal conductivity coefficient *λ* and the thermal resistance R’.

The approach for determining the thermal parameters relied on measuring the electrical voltage and translating it into heat flux density using Equation (1) provided by the device manufacturer:

(1) $$q=\frac{Uqc}{0.0103}$$

where *q* is the heat flux density, [W/m^2^]; and Uqc is the voltage of the flowing current, [mV].

At the same time, the temperatures of the top (hot) and bottom (cold) surfaces of the samples on the test bench were recorded together with the air temperatures inside and outside the cold chamber. These measurements were made using type K thermocouples. Using the recorded temperatures and the heat flux density during the steady-state phase of heat transfer through the sample, the heat transfer coefficient was determined using Equation (2):

(2) $$\lambda =\frac{d\cdot q}{T_{g}-T_{d}}$$

where *λ* is the thermal conductivity of the material, W/(m·K); *d* is the thickness of the test sample, m; *q* is the heat flux density, W/m^2^; *T_g_* is the temperature of the upper surface of the sample, °C; and *T_d_* is the temperature of the lower surface of the sample, °C.

#### 2.4.2. Compressive Strength and Quasi-Static Indentation Strength

The compressive strength tests were performed according to the PN-EN ISO 604 [39] standard using a TINIUS OLSEN H25KT testing machine. Five specimens of each material were subjected to compression at a speed of 2.0 ± 0.25 mm/min. The samples were deformed to 15% to allow for the potential evaluation of the materials’ behavior after reaching the yield point. Compressive strength was determined as the maximum stress. The modulus of elasticity was calculated as the slope of the linear portion of the stress–strain curve.

**Figure 5:**
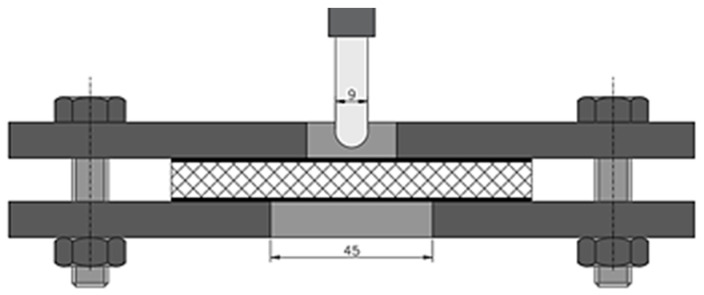
Schematic of the quasi-static punch shear test apparatus.

The quasi-static penetration test was carried out using a TINIUS OLSEN H25KT testing machine in accordance with the ASTM D-732 norm [40]. The punch test scheme is shown in Figure 5. A rounded cylindrical punch of 9 mm diameter was used. The samples, 50 mm × 50 mm, were placed between two metal plates with centrally located holes. The diameter of the hole in the support plate was 45 mm. The QSPT was carried out with a constant displacement rate of 1.25 mm/min and a total displacement of 20 mm. The QSPT tests were conducted to determine the maximum force (*F_max_*) and to calculate the punch shear strength (*PSS*) of the analyzed structures. The *PSS* value corresponds to the maximum force counteracting the deformation of the material due to penetrator pressure and thus its failure resistance.

Based on the collected data (averaged from five samples for each material), the following parameters were calculated:

- Energy absorbed by the material (*E_a_*) refers to the total amount of energy that the material was able to absorb during the puncture process. It is the work performed by the penetrating force to cause damage to the material. The value of the absorbed energy was determined by integrating the area under the force-displacement curve:

(3) $$E_{a}=\int_{0}^{20}f\left(x\right)dx$$

where for integral limits, 0–20 mm is the displacement range of the punch; and *f*(*x*) is a function describing the relationship between the force and the displacement (position) of the punch.

- Punch Shear Strength (*PSS*) is an index used to assess the ability of a material to resist punching forces:

(4) $$PSS=\frac{F_{max}}{\pi \cdot \delta \cdot H_{c}}$$

where *F_max_* is the maximum punching force, N; *δ* is the punch diameter, mm; and *H_c_* is the sample thickness, mm.

- Specific Energy Absorption index (*SEA*) is a measure that determines the amount of energy absorbed by a unit of mass of a material:

(5) $$SEA=\frac{E_{A}}{m}$$

where *E_A_* is the energy absorbed by the sample, J; and *m* is the sample mass, kg.

## 3. Results and Discussion

Statistical analyses were performed using STATISTICA 13 (TIBCO Statistica, Palo Alto, CA, USA). A significance threshold of *p* ≤ 0.05 was used for the analysis in accordance with standard practice in thermal materials testing. Table 4 shows the determined values of the thermal conductivity coefficient (*λ*) and the thermal resistance (R), while Table 5 presents the results of the compressive strength and puncture resistance tests of the proposed samples produced using the FDM technology.

The significance of the effect of the porosity of the inner core of the manufactured composites and the height of the cells in their structure on the physical properties was then assessed. A multivariate analysis of variance (ANOVA) was used to determine this effect. The results obtained are presented in Table 6, with significance levels (*p*-values) in the last column of the table. Values less than 0.05 indicate a significant effect of porosity and cell size on the coefficient of thermal conductivity (*λ*, W/(m-K)), the coefficient of thermal resistance R, (m^2^·K)/W, and the compressive strength (CS) and tensile strength (*PSS*). In addition, the analysis showed (F-values for each dependent variable) that the highly dominant factor relative to the other input factors is the cell porosity (P) of the composites. Furthermore, each input factor is optimized independently of the others.

### 3.1. Thermal Conductivity and Thermal Resistance

**Figure 6:**
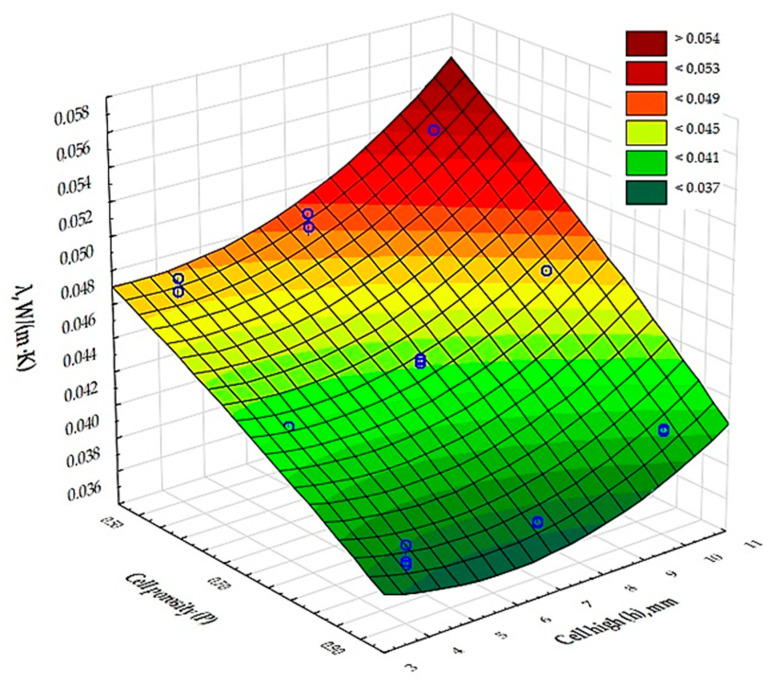
Interpretation of experimental data determining the influence of input factors (independent variables) on the value of the thermal conductivity coefficient (*λ*) of the composite.

**Figure 7:**
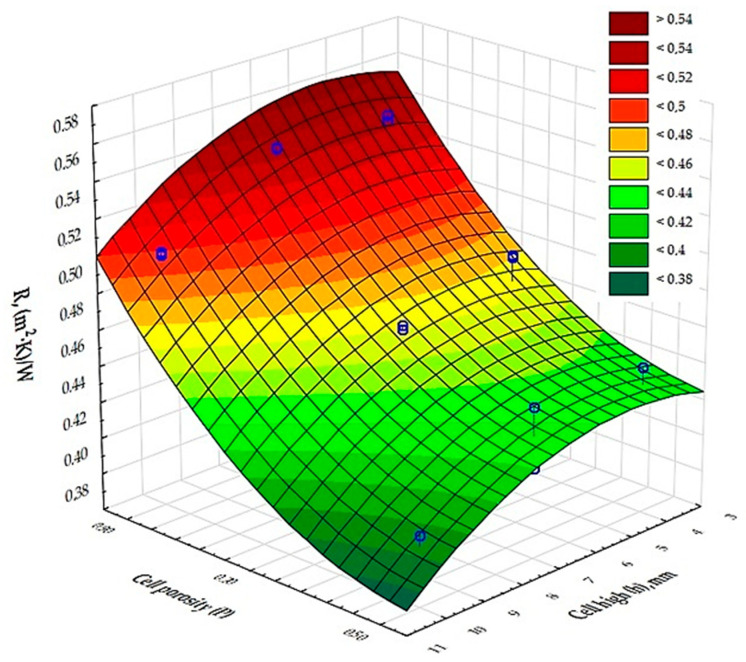
Interpretation of experimental data determining the influence of input factors (independent variables) on the value of the thermal resistance coefficient (R) of the composite.

Figure 6 and Figure 7 show the results of the measurement of the thermal conductivity and thermal resistance coefficients of the test samples marked 1–9.

The results of the analysis of variance, presented in Table 6, show that there is an effect of cell porosity (P) and cell size (h) on the values obtained for the coefficients of thermal conductivity and thermal resistance. Figure 6 and Figure 7 show a graphical comparison of the results obtained for the different types of cell porosity in the composite on which the experiment was carried out. Samples with higher porosity, of the order of 90%, had a lower thermal conductivity and therefore a higher thermal resistance coefficient than samples with lower porosity, i.e., 50% and 70%, and the difference in the results obtained was large. This shows that the 90% porosity composite had better insulating properties. It was also shown that the cell size (h) in the composite has a significant effect on the thermal conductivity coefficient and thermal resistance. Figure 6 and Figure 7 show the resulting difference between the air cell size parameters in a cellular structure based on the Kelvin cell shape. Samples with the smallest cell height of 4 mm had a lower thermal conductivity coefficient (*λ*) and thus a higher thermal resistance coefficient than samples with larger cell heights, i.e., 7 mm, 10 mm. The difference in the values obtained was large in this case. This shows that the composite with a cell height of 4 mm had better insulating properties.

Analysis of measurements of composites printed from PLA with an inner core with a Kelvin cell structure suggests that this composite has comparable thermal insulation properties to structures produced using other 3D printing technologies [36,41,42]. Thermal conductivity and thermal resistance coefficients for 90% porosity monolayer samples of PLA with Kelvin cell diameters ranging from 4 mm to 10 mm were in the range of 0.037–0.052 W/(m⸱K) and 0.40–0.54 (m^2^·K)/W, while for composites made by SLS technology from grey PA12 [42] and a porosity of 90% with a cell diameter of 4 mm to 10 mm they are 0.047–0.59 W/(m⸱K) and 0.34–0.43 (m^2^·K)/W. However, according to [36], for barriers produced by SLA technology with a porosity of 95% and a cell diameter of 6 mm—using different UV resins—the value for thermal conductivity was between 0.042 and 0.053 W/(m⸱K), while for thermal resistance it was between 0.38 and 0.59 (m^2^·K)/W. On the basis of the analysis carried out, it can be concluded that composites with the parameters described above do not differ significantly in their physical properties, regardless of the 3D printing technology used, which means that they are very good thermal insulation materials.

### 3.2. Compressive Strength

The results of the analysis of variance presented in Table 6 show that there was an effect of cell porosity (P) and cell size (h) on the compressive strength values obtained. In addition, the input variable composite porosity (P) was shown to be a highly dominant factor over the other input factors.

**Figure 8:**
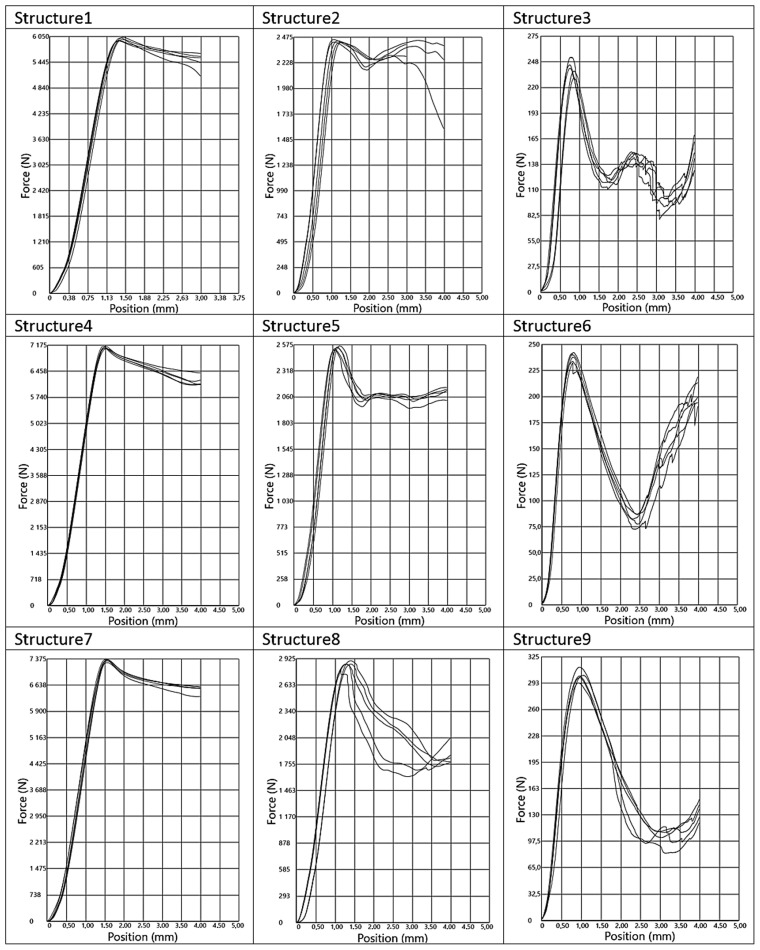
Compressive force measurement curves for samples 1–9.

Plots of force versus punch position for all specimens tested are shown in Figure 8. Three different types of compression behavior of the specimens can be observed. The maximum force occurs at a deformation of approximately 7.5% (1.5 mm) for specimens 1, 4, and 7, corresponding to a compressive force of approximately 6.0–7.4 kN. Above this threshold, there is a change in force of approximately 10% as the strain increases to 20%, at which point the test ends.

The maximum force of 2.5–2.9 kN is observed at a deformation of approximately 5% (1.0 mm) when specimens 2, 5, and 8 are compressed. Next, the force decreases rapidly by 10, 20, and 40% respectively as the deformation increases to 10% (2.0 mm), and a plateau region is observed.

**Figure 9:**
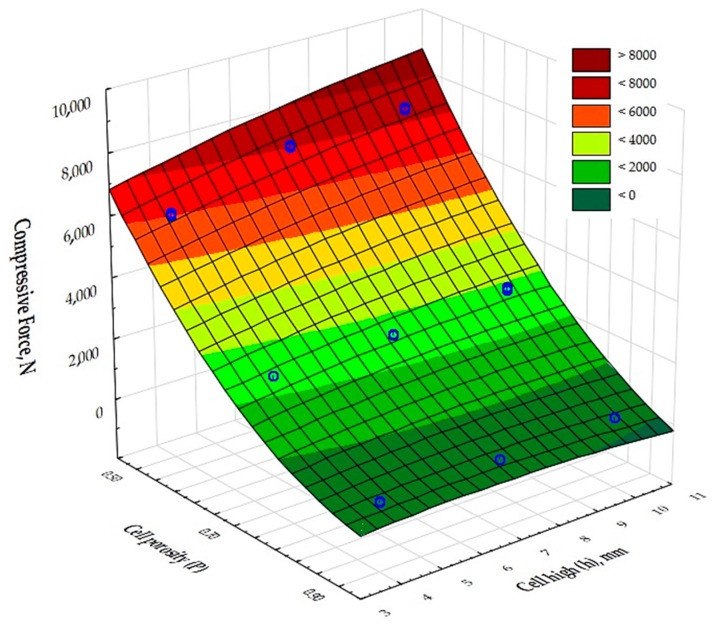
The values of the maximum compressive forces for the test specimens 1–9.

A deformation of 5% is observed when specimens 3, 6, and 9 are compressed, corresponding to a maximum force of 230–290 N. The force then decreases by 50% (system 3), 70% (systems 6 and 9), and reaches a minimum at a deformation of 10–15% (2–3 mm). Further, the force value starts to increase rapidly, which is probably related to the damaged internal structure of the samples. The averaged values of the maximum compressive force are summarized in Figure 9.

**Figure 10:**
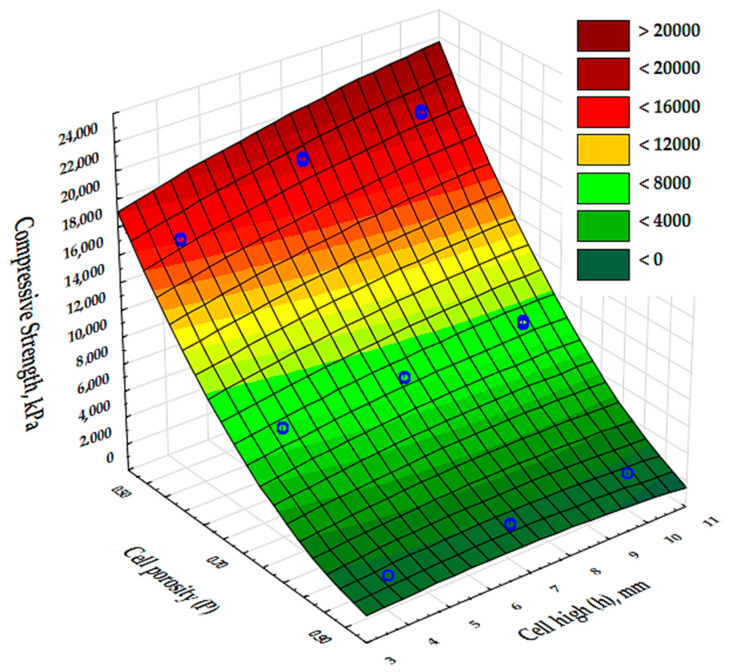
The values of the compressive strength for the test specimens 1–9.

Figure 10 shows a graphical comparison of the results obtained for the different types of cell porosity in the composite on which the experiment was performed. Samples with higher porosity, of the order of 90%, obtained lower compressive strength values than samples with lower porosity, i.e., 50% and 70%, and the difference in the results obtained was large. This shows that the 50% porosity composite had the best compressive strength properties. It was also shown that the size of the cells (h) in the formed structures had little significant effect on the compressive moduli of the samples.

**Figure 11:**
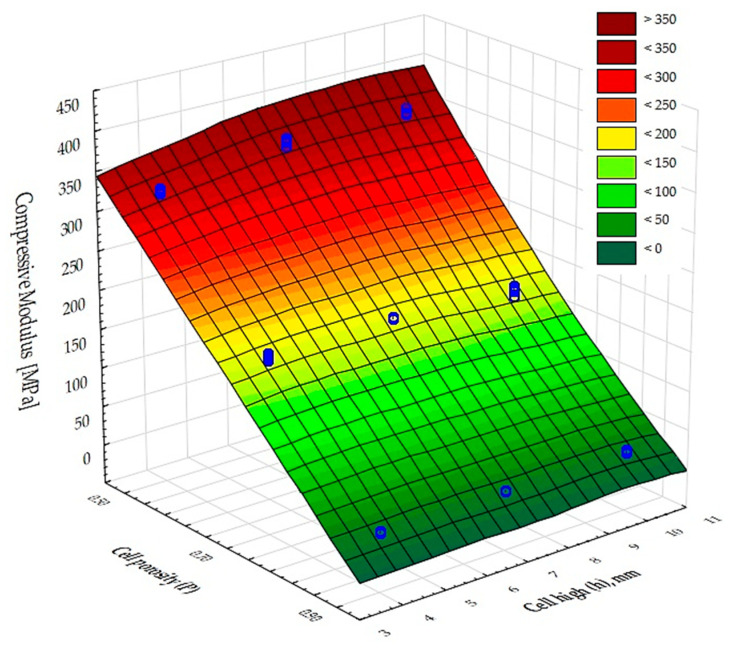
Compressive modulus values for the test samples.

**Figure 12:**
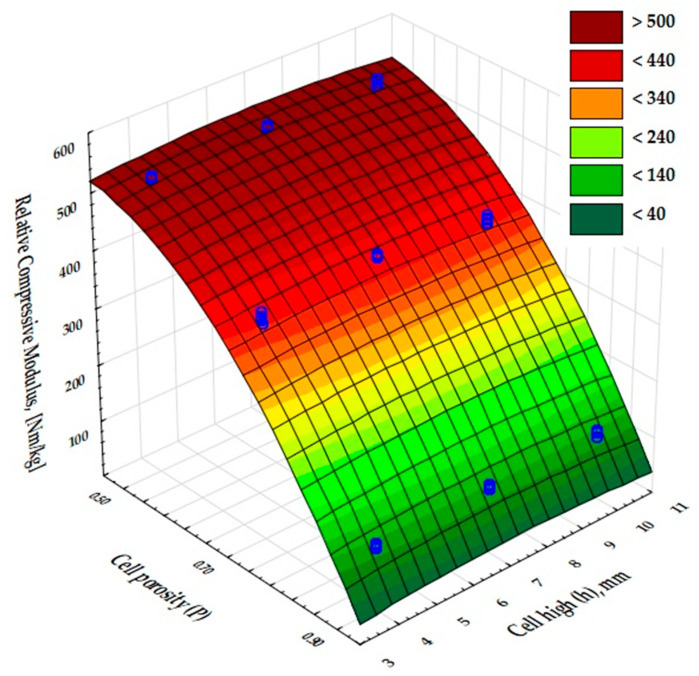
Relative compressive modulus for samples 1–9.

The moduli of compressibility of the samples determined from the graphs (Figure 8) are shown in Figure 11. The values for samples 1, 4, and 7 are similar and amount to about 350 MPa; for samples 2, 5, and 8 they are twice as low and equal to 175 MPa; while the systems 3, 6, and 9 show a modulus of about 26 MPa. However, in the case of incompletely filled samples, such as those obtained by FDM 3D printing technology, it seems more appropriate to relate the strength parameters to the apparent density of the samples obtained. The values of the relative compressive moduli calculated in this way are shown in Figure 12. It can be seen that the compressive moduli related to density are comparable for systems 1, 4, and 7 as well as 2, 5, and 8 and differ by only about 25%.

**Figure 13:**
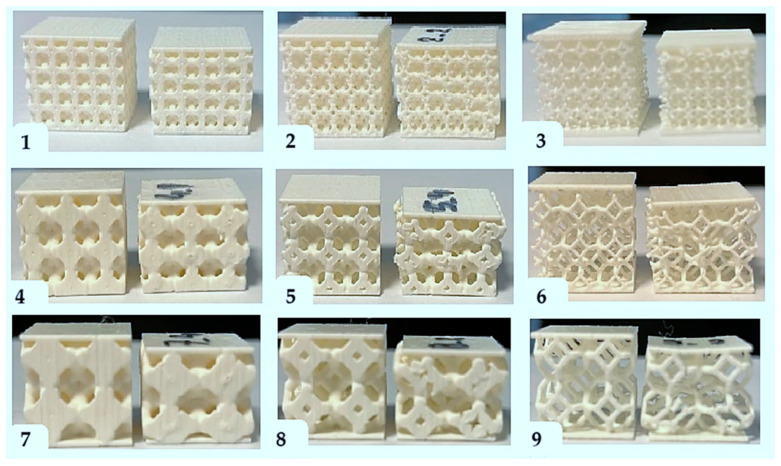
Images of the samples before and after compressive strength determination.

Images of the specimens before and after compressive strength determination are shown in Figure 13. Structures 1, 4, and 7 exhibited minimal internal structural damage (plastic deformation) after exceeding the yield strength. Structures 2 and 5 demonstrated similar behavior. In contrast, structures 3, 6, 8, and 9 suffered significant internal structural damage. Systems 6 and 9, in particular, experienced severe deformation during the compression test, which is attributed to their low fill ratio.

### 3.3. Quasi-Static Indentation Strength

The results of the analysis of variance, presented in Table 6, show that there was an effect of cell porosity (P) and cell size (h) on the quasi-static indentation strength values obtained. In addition, the input variable composite porosity (P) was shown to be a highly dominant factor over the other input factors.

**Figure 14:**
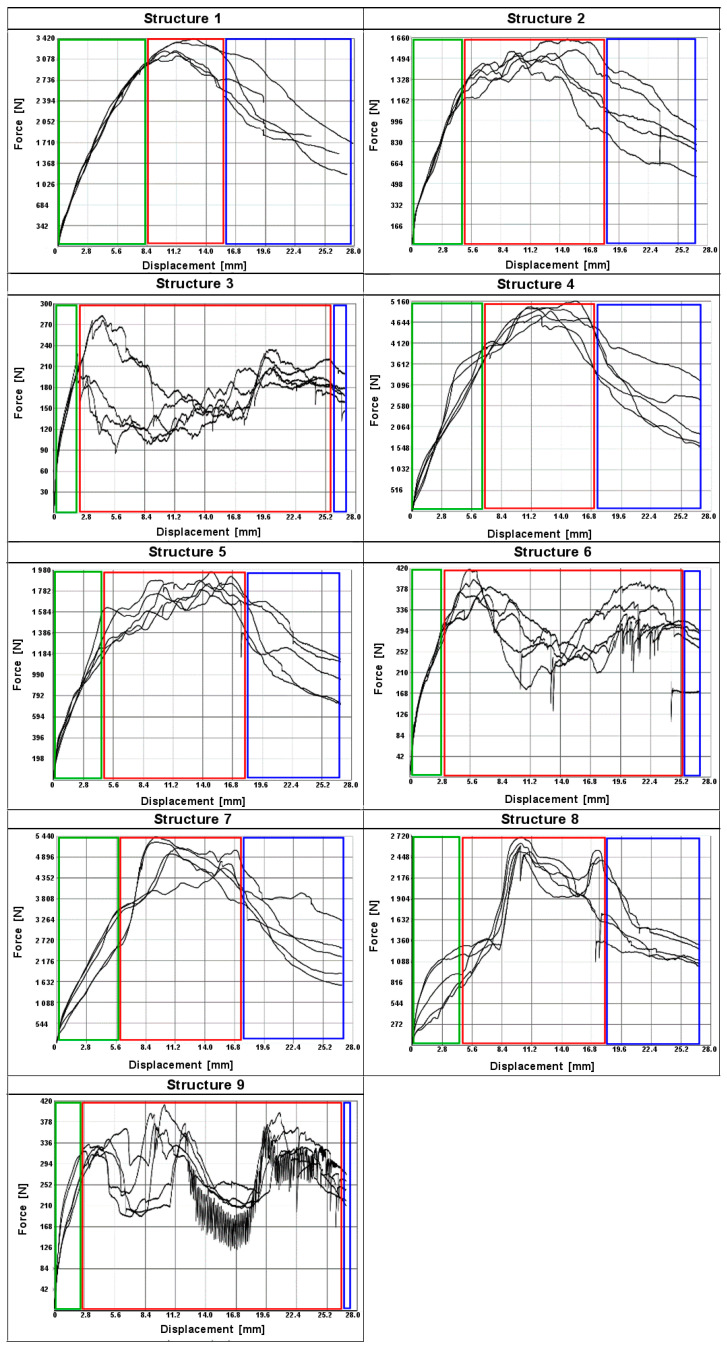
Punch force measurement curves for systems 1–9.

The results of the punch force measurements on structures 1–9 are shown in Figure 14. The failure process of the tested structures in quasi-static indentation strength can be divided into stages corresponding to three areas on the force-displacement curves obtained during the tests. These areas are the elastic deformation phase (I), the rapture phase (II), and the dynamic friction phase (III). These phases are indicated in the figure by the colours green, red, and blue, respectively.

The force-displacement graphs for systems 1, 4, and 7 are the most symmetrical and smooth. The maximum is usually only at a displacement of about 10–12 mm, indicating the fracture of a solid internal structure. These systems have the highest density (the determined bulk density is 624, 665, and 661 kg/m^3^, respectively), and the highest maximum force value is observed here (3.5–5.5 kN).

We have several local force maxima (structure 8—two maxima) in systems 2 and 5, indicating damage to other existing structures within the sample during the puncture process in the displacement range of 4–18 mm. Here the maximum forces are slightly lower and are around 1.6–2.7 kN. However, the apparent density of structures 2, 5, and 8 is slightly lower at 453, 441, and 448 kg/m^3^.

Systems 3, 6, and 9 have the lowest apparent densities of 230, 221, and 228 kg/m^3^, respectively. They have different local extrema on the force-displacement diagram. On average, they have two maxima at displacements of 5 and 20 mm. They have a minimum at a displacement of about 15 mm. The maximum forces in these systems are small at 300–400 N.

**Figure 15:**
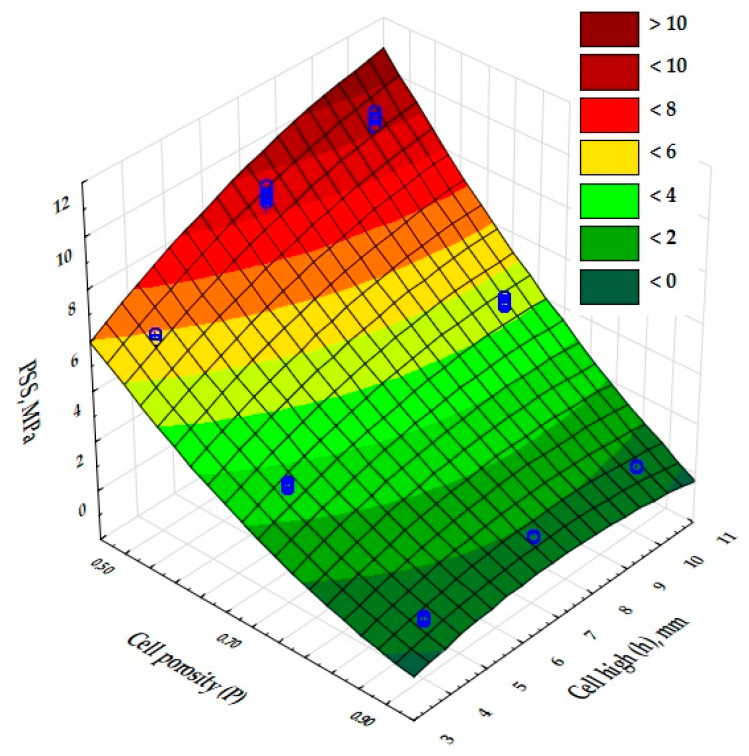
Results of the *PSS* measurements for the tested structures 1–9.

In order to fully interpret the above results, the *PSS* (punch shear strength) was calculated. The values obtained are shown in Figure 15. The highest punch shear strength value, 9 MPa, was recorded for system 7, with a slightly lower *PSS* value shown for system 4 (8.8 MPa). Sample 1 has a *PSS* value of approximately 5.7 MPa. It was observed that for the same porosity of 0.5, increasing the height of the inner core cells in the composite from a value of 4 mm (sample 1) to 9 mm (sample 7) to 7 mm (sample 4) had a positive effect on the test result. Systems 8, 5, and 2 had lower *PSS* values of 4.6 MPa (9 mm high cell), 3.3 MPa (7 mm high cell), and 2.8 MPa (4 mm high cell) respectively. The above values were obtained for samples with a constant porosity of 0.7 and increasing cell size from h = 4 mm to 9 mm. The best *PSS* value was obtained for the composite with a porosity of 0.90, whereas the puncture strength of samples 3 (cell height 4 mm), 6 (cell height 7 mm), and 9 (cell height 9 mm) did not exceed 1 MPa, and the values obtained were close to each other.

These results show the significant influence not only of the porosity of the printed composites but also of the cell size obtained in the samples tested.

**Figure 16:**
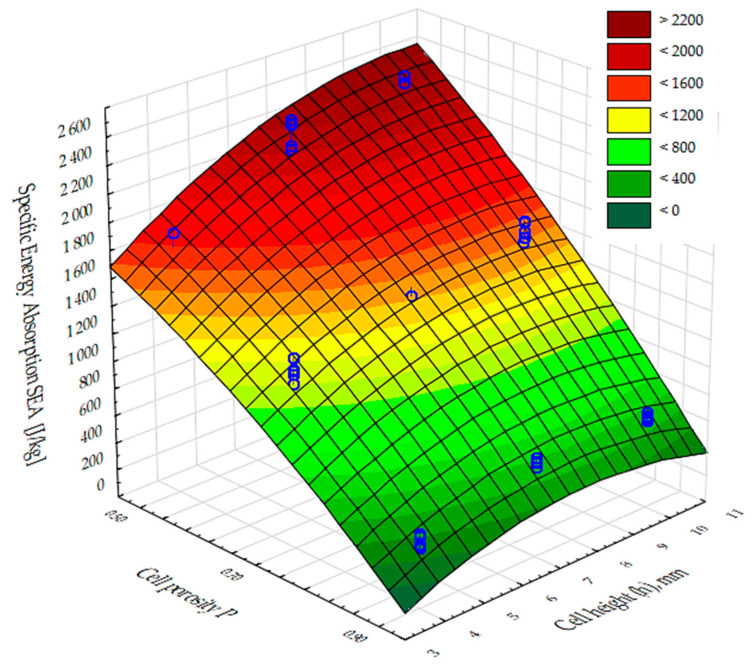
SEA results for test samples 1 to 9.

The results of the calculation of the specific energy absorbed (SEA) during the run-through test are shown in Figure 16. The highest energy value (approximately 2.1 kJ/kg) was recorded for samples 4 and 7, which is approximately 40% higher than sample 1 (approximately 1.5 kJ/kg). Rather high values of absorbed energy were also observed for systems 8, 5, and 2 (1.1–1.4 kJ/kg). The lowest values of absorbed energy at breakthrough were achieved by systems 3, 6, and 9 (300–500 J/kg). The obtained calculation results confirm the significant influence of porosity and cell diameter size on the composite. The lower the porosity and the larger the cell diameter, the higher the forces obtained.

**Figure 17:**
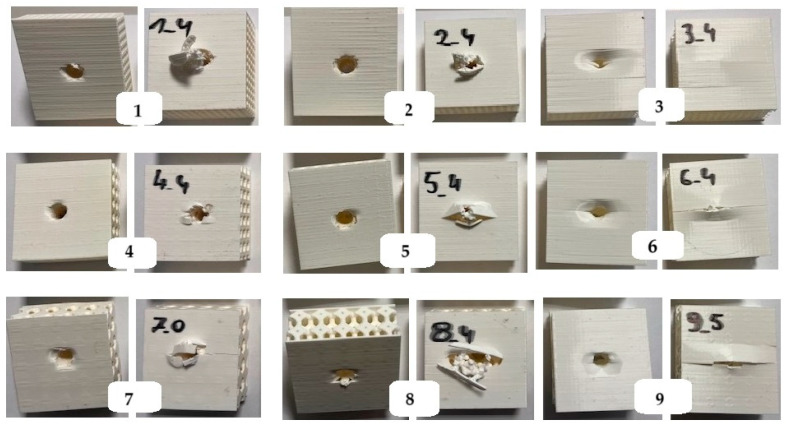
Damage patterns across composite samples.

The damage process of tested samples 1–9 occurs in stages, distinguishing both intra-layer damage and inter-layer defects due to the complexity of the structures. The aim of the visual analysis of the post-puncture systems was to identify and assess the damage that had occurred. Figure 17 shows images of the post-puncture samples. The effectiveness of the structures in absorbing energy is closely related to the porosity of the composite samples. Phenomena resulting from puncture overstrength include both delamination and matrix cracking. In addition, damage can occur as a result of friction between the punch and the sample.

Mechanical tests performed by researchers [43] on porous polylactic acid (PLA) structures that were 3D printed from a Kelvin model for bone tissue engineering applications also showed that increasing porosity decreases the mechanical properties of the scaffold. While increasing cell size at constant porosity increases Young’s modulus and yield stress, it also decreases the flexibility and strength of the scaffolds. Other authors [44] used FEM modeling to show that the yield stress increases rapidly as the relative density of the lattice structure increases. The authors of [45] carried out experimental studies on porous polylactic acid (PLA) structures 3D printed from the Kelvin model. According to them, the mechanical properties of Kelvin model lattices are influenced by (1) elementary cell size, (2) fill density, (3) material, and (4) layer thickness. In their study, they kept the elementary cell size and the shell thickness constant, while the fill density was the only variable parameter. A Kelvin cell lattice with three different fill densities was considered, and the authors assumed a layer thickness of 2 mm, slightly higher than the standard thickness in the range of 0.5 mm to 1.5 mm, in order to verify the role that layer thickness plays in energy absorption. Finally, the researchers showed that Young’s modulus and stress increase with increasing fill density. It was observed that coating thickness increases the elastic region and energy absorption capacity of the component. Yield strength and ultimate stress increase with increasing lattice fill density. A lattice structure with 20% infill density is preferred by the industry for its lightness and high strength. A 20% infill density also saves on material consumption. It has been observed that a Kelvin cell with a 20% infill density absorbs 2.81 MJ/m^3^ of energy.

## 4. Conclusions

The aim of this paper is to develop an innovative thermal insulation barrier based on a Kelvin foam model, fabricated using 3D FDM printing technology, and to experimentally determine the thermo-mechanical properties of the barrier (density, thermal conductivity, thermal resistance, compressive strength, and quasi-static indentation strength) as a function of the variables determining its internal geometry.

The study showed that the single-layer cellular structures achieved thermal conductivity coefficients below 0.06 W/(m·K), demonstrating their very good thermal insulation properties, comparable to most standard materials used in construction. In addition to the thermal properties, the authors also determined the basic mechanical properties of the produced cellular materials, i.e., the compressive and puncture strength.

On the basis of the research carried out, the authors of the paper indicate that

- The two-factor ANOVA analysis of variance showed a significant effect of porosity (P) and cell height (h) in the inner core of the composite on the thermal conductivity coefficient (*λ*), thermal resistance (R), compressive strength (CS) and punch shear strength (*PSS*);
- The input variable composite porosity (P) was shown to be highly dominant over the other input factors;
- The values of the thermal conductivity coefficient (*λ*) of all the tested samples were between 0.037 and 0.052 W/(m·K), which is lower than the maximum value (0.065 W/(m·K)) required for insulating materials according to PN-EN 2020-12 [46]. This means that the obtained composites meet the thermal efficiency standards;
- The best insulation results were obtained for single-layer composites with a cell height of 4 mm and a porosity of 90%, where the coefficient of thermal conductivity was 0.038 W/(m-K) and the thermal resistance was 0.537 (m^2^·K)/W;
- The density-dependent compressive moduli for samples 1, 4, and 7 (P = 50%) and 2, 5, and 8 (P = 70%) are comparable and differ by only about 25%;
- The compressive moduli for samples 1, 4, and 7 are almost the same at about 350 MPa, while the moduli for samples 2, 5, and 8 are twice as low at 175 MPa, and samples 3, 6, and 9 (P = 90%) have a modulus of about 26 MPa;
- The size of the cells in the fabricated structure has a significant effect on the puncture resistance value. The highest puncture resistance value of 9 MPa was recorded for sample 7, a slightly lower *PSS* value of 8.8 MPa was recorded for sample 4, while sample 1 had a *PSS* value of approximately 5.7 MPa;
- The porosity (P) of the composite structure has a significant effect on the puncture resistance. The lowest values were obtained for samples with 90% porosity; the value for all three samples, 3, 6, 9, did not exceed 1 MPa.

In conclusion, the authors’ task was to determine the effect of cell size (h) and porosity (P) parameters of PLA structural frameworks produced using Kelvin model-based 3D FDM printing technology in terms of their use in heat transfer technology. As a result of the test, it was shown that the resulting 3D cellular composites offer an innovative and environmentally friendly approach to thermal insulation. Therefore, as part of further research, the authors focused on the introduction of natural fiber admixtures into the composition of the PLA filament in order to improve the mechanical properties of the cellular structures produced from it, mainly for composites with a porosity of 90%, which were characterized by the best thermal properties.

## Figures and Tables

**Table 1:** Coding of independent variables.

Coded Levels	Cell Porosity P	Cell Height h, mm
−1	0.50	4
0	0.70	7
1	0.90	10

**Table 2:** Experimental matrix of (3^2^) full factorial design for independent variables.

Experiment Code	Cell Porosity P	Cell Height h, mm
1	0.50	4
2	0.70	4
3	0.90	4
4	0.50	7
5	0.70	7
6	0.90	7
7	0.50	10
8	0.70	10
9	0.90	10

**Table 3:** 3D printing parameters.

Parameter	Value
Nozzle diameter	0.4 mm
Layer thickness	0.16 mm
Extrusion temperature	230 °C
Print speed	Up to 40 mm/s
Infill density	100%
Nozzle diameter	0.4 mm

**Table 4:** Descriptive statistics of the dependent variables: thermal conductivity coefficient (*λ*) and thermal resistance coefficient (R) for specimens produced by the 3D FDM additive method. M—mean, Me—median, Min—minimum, Max—maximum, SD—standard deviation.

Variability	Valid N	M	Me	Min	Max	SD
*λ*, W/m·K	27	0.044	0.043	0.037	0.052	0.005
R, K·m^2^/W	27	0.463	0.459	0.399	0.537	0.048

**Table 5:** Descriptive statistics of the dependent variables: compressive strength (CS), punch shear strength (*PSS*), for specimens produced by the 3D FDM additive method. M—mean, Me—median, Min—minimum, Max—maximum, SD—standard deviation.

Variability	Valid N	M	Me	Min	Max	SD
CS, kPa	45	8075	6380	577	18,400	6914
*PSS*, MPa	45	3.9	3.3	0.4	9.6	3.2

**Table 6:** A quantitative assessment of the main effects—the identification of the impact of dominant and statistically significant input factors on the dependent variable thermal conductivity coefficient (*λ*) and thermal resistance coefficient (R), compressive strength (CS) and quasi-static indentation strength (*PSS*) for single-layer composite specimens (SS—sum of squares, df—degrees of freedom, MS—mean square, F—F ratio, p—significance level (*p*-values)).

Symbol That Identifies the Input Factors	df	SS	MS	F	p
*λ*, W/(m·K)					
absolute term	1	0.052	0.052	53,581.2	0.000
P	2	0.0005	0.00024	247.9	0.000
h	2	0.0001	0.00005	48.95	0.000
error	22	0.00002	0.000001		
general	26	0.0006			
R, (m^2^·K)/W					
absolute term	1	5.7912	5.79116	69,336.75	0.000
P	2	0.05153	0.0258	308.49	0.000
h	2	0.00768	0.0038	45.99	0.000
error	22	0.00184	0.0001		
general	26	0.06105			
CS, GPa					
absolute term	1	2934.06	2934.06	0.007	0.000
P	2	2068.103	1034.05	0.0023	0.000
h	2	17.865	8.93	2.064 × 10^−5^	0.000
error	40	17.307	0.432		
general	44	2103.276			
*PSS*, MPa					
absolute term	1	717.86	717.86	1469.1	0.000
P	2	395.21	197.60	404.4	0.000
h	2	26.05	13.02	26.6	0.000
error	40	19.55	0.49		
general	44	440.80			

## Data Availability

Data is contained within the article.
